# Using the theory of planned behavior to predict factors influencing fast-food consumption among college students

**DOI:** 10.1186/s12889-023-15923-1

**Published:** 2023-05-27

**Authors:** Maryam Sajjad, Afifa Bhatti, Barry Hill, Basem Al-Omari

**Affiliations:** 1 Faculty of Allied Health Sciences, University of South Asia, Lahore, Pakistan; 2 College of Medicine and Health Sciences, Khalifa University, Abu Dhabi, United Arab Emirates; 3 Department of Nursing, Midwifery, and Health, University of Northumbria, Newcastle Upon Tyne, UK; 4 Department of Epidemiology and Population Health, College of Medicine and Health Sciences, Khalifa University, Abu Dhabi, United Arab Emirates

**Keywords:** Fast food, Behavioral intention, Subjective norm, Perceived behavior, Adolescents, Structural equation modeling

## Abstract

**Purpose:**

The purpose of this research is to examine the behavioral factors that impact fast food consumption (FFC) among college students in Pakistan by applying the theory of planned behavior (TPB).

**Methods:**

Cross-sectional survey was distributed to college students in Pakistan. The questionnaire examines the factors associated with six categories: demographics, FFC pattern, intention for FFC, attitudes toward FFC, Subjective Norms (SN), and Perceived Behavioral Control (PBC). Data analysis was conducted using SPSS and SPSS AMOS software using descriptive statistics, inferential statistics (chi-square, t-test, Pearson correlation, and multiple regression analysis), and structural equation modeling (SEM) Analysis.

**Results:**

A total of 220 questionnaires were completed (97 males and 123 females). There were significant differences in FFC association with gender. Among the constructs of TPB, behavioral intention (BI) and SN are the strongest predictors of the FFC (*p* < .05). TPB has significantly predicted FFC behavior with a variance of R^2^ 0.603. The SEM analysis shows that the data collected were incompatible with the theoretical TPB model, making it unfeasible to test our five hypotheses or interpret the results due to the poor fit of the model with the data.

**Conclusions:**

To ensure a good fit of the data with the specified TPB model in SEM analysis, the number of indicators should be limited (≤ 30), or the sample size should be greater (N ≥ 500). Pakistani college students' FFC is mainly influenced by friends and the increased popularity of fast food, despite their knowledge of its negative health effects. Educational programs should target specific harmful effects of fast food, and SN and BI are the strongest predictors of FFC among TPB constructs. These findings can be useful for developing targeted interventional health strategies and future research.

**Supplementary Information:**

The online version contains supplementary material available at 10.1186/s12889-023-15923-1.

## Introduction

Recently, fast-food restaurants are often seen as a special treat for children [1], which contributes to the increasing prevalence of obesity and other food-related diseases [1, 2]. In 2016, more than 340 million children and young adults aged 5 to 19 were overweight or obese [3]. In 2020, 39 million children under the age of 5 were diagnosed as obese or overweight [4]. Since 1975, global obesity has almost tripled, with more than 1.9 billion adults over the age of 18 being overweight and over 650 million being obese [5].

Additionally, there are now several identified non-communicable diseases directly linked to fast food-based diets [6]. These include obesity, cardiovascular disease, type 2 diabetes mellitus, hypertension, and an increased chance of stroke [7]. A study has reported a clear correlation between regular fast-food consumption (FFC) and weight gain as well as an increased risk of insulin resistance over 15 years. Individuals who ate at fast-food restaurants more than twice a week gained an average of 4.5 kg more weight and experienced a 104% greater increase in insulin resistance compared to those who ate less than one fast-food meal per week, both at the beginning and end of the study [8].

Adolescents have a high tendency to consume energy-dense fast food products, leading to significant scientific research in this area [1]. This preference for fast food is particularly prevalent in low- and middle-income countries such as Pakistan and is often viewed as a nutritional transition from traditional diets to a more Westernized diet, which is characterized by excessive consumption of high-fat and processed foods, as well as sugary drinks and sweets [9, 10]. The cultural shifts of FFC in Pakistan are driven by the normalization of fast-food diets among young adults and adolescents, the convenience of Western fast-food options, competitive pricing, and the use of celebrities in marketing [11]. Other themes include the influence of peer pressure and fast food as a quick solution for busy parents [12, 13]. This is leading to the increasing popularity of fast food, which is currently growing at a rate of 20% per year in Pakistan [14].

The burden of obesity in developing countries, including Pakistan, has increased significantly. The increased availability of fast food and increased sedentary lifestyles of people have resulted in ¼ of Pakistan’s population now being obese [15, 16]. In 2018, it was reported that 16.2% of students studying in Pakistan were obese and 15% were overweight [16], indicating Pakistan’s younger generation is at heightened risk of premature death and ill health due to obesity-related complications. Male adolescents are those identified as making poorer choices when consuming fast foods [17, 18].

### Literature review

Assessing and evaluating fast food intake among adolescents and young adults is crucial in detecting factors associated with overweight and obesity [19]. Identifying influential factors such as food preferences, family eating patterns, and social norms can guide interventions promoting healthy eating behaviors [20]. The Theory of Planned Behavior (TPB) is a well-known model that predicts eating behavior based on norms and beliefs related to fast food and snack consumption [21, 22]. Derived from the theory of reason action (TRA), TPB explains health behaviors that are not entirely under an individual’s control. Furthermore, TPB allows a good understanding of people’s attitudes and intentions about their food choices [23]. It is also noted that perceived behavioral control (PBC) is the most significant aspect of TPB [24]. The TPB details how the influences upon an individual determine their decisions to follow a particular behavior. Furthermore, TPB is effective as it includes these main constructs: attitudes, subjective norms (SN), PBC, and intentions [21, 23].

According to the TPB, ‘attitude’ is a person's positive or negative evaluation of a particular outcome related to a behavior, such as eating fast food [25]. Attitude has two components: affective attitude, which reflects a person's emotions, and cognitive attitude, which reflects their knowledge or beliefs. Positive attitudes are more likely to support behavioral intention (BI), while negative attitudes are more likely to hinder it [26, 27]. The concept of PBC encompasses two dimensions perception of control and self-efficacy [28]. Perception of control relates to external factors such as accessibility, task difficulty, and others' behavior, while self-efficacy involves internal factors like motivation, ability, and personality [29]. The TPB postulates that both dimensions of PBC facilitate the formation of (BI)for positive actions and impede the intention for negative actions. SN refers to an individual's perception of social influence that either encourages or discourages them to engage in a particular behavior. These perceptions are based on the attitudes of significant individuals, known as referents, who approve or disapprove of the behavior in question [30].

The TPB predicts and explains a range of health behaviors and intentions including smoking, drinking, health services utilization, breastfeeding, and substance use. The constructs of TPB collectively represent a person's actual control over the behavior [31]. According to the TPB, a person’s desire for action will be higher if they have more favorable views about what they are undertaking, their PBC, and the SN. Stronger intention, alongside having perceived behavior control, indicates a higher likelihood of completing the behavior [32].

A recent systematic review of food choices has shown that attitudes of people are strongly correlated with intention, SN, and PBC, and intention has a strong association with behavior compared with PBC [33]. Current available literature tends to explore TPB in high-income western countries, with limited data available for lower-income countries. Therefore, there are several considerations to be handled by researchers due to the uniqueness of applying such research to a lower-income country such as Pakistan. For instance, socioeconomic status represents the underlying basic cause of malnutrition and is composed of multiple variables such as type of sanitation facilities, source of drinking water, and housing infrastructure [34, 35]. Poor socioeconomic status is directly related to illiteracy, unemployment, reduced purchasing power, and poor health and nutritional outcomes [36]. Studies conducted in China, Mexico, and Sub-Saharan African countries have shown a higher risk of coexistence of stunting with overweight/obesity in children of the low socioeconomic class than in children of higher socioeconomic class. Additionally, in Pakistan, the double burden of malnutrition is becoming increasingly apparent, with almost one in three children underweight (28.9%) alongside a high prevalence of overweight (9.5%) [37].

In consideration of such differences in Pakistan compared to high-income westernized FFC, it is unclear if a model that predicts some parameters connected to FFC will work in lower-income countries. As a result, it appears that testing the models' utility in each group is required. This study looks at determinants of fast-food intake among college students in Pakistan, utilizing the TPB model to improve the predictability of intention and behavior. Furthermore, this study will add more literature on the FFC habits of students in a highly densely populated country such as Pakistan. Therefore, the purpose of this research is to examine the behavioral factors that impact FFC among college students in Pakistan by applying TPB. The results would help plan nutrition intervention programs.

### Hypothesis development

**Fig. 1:**
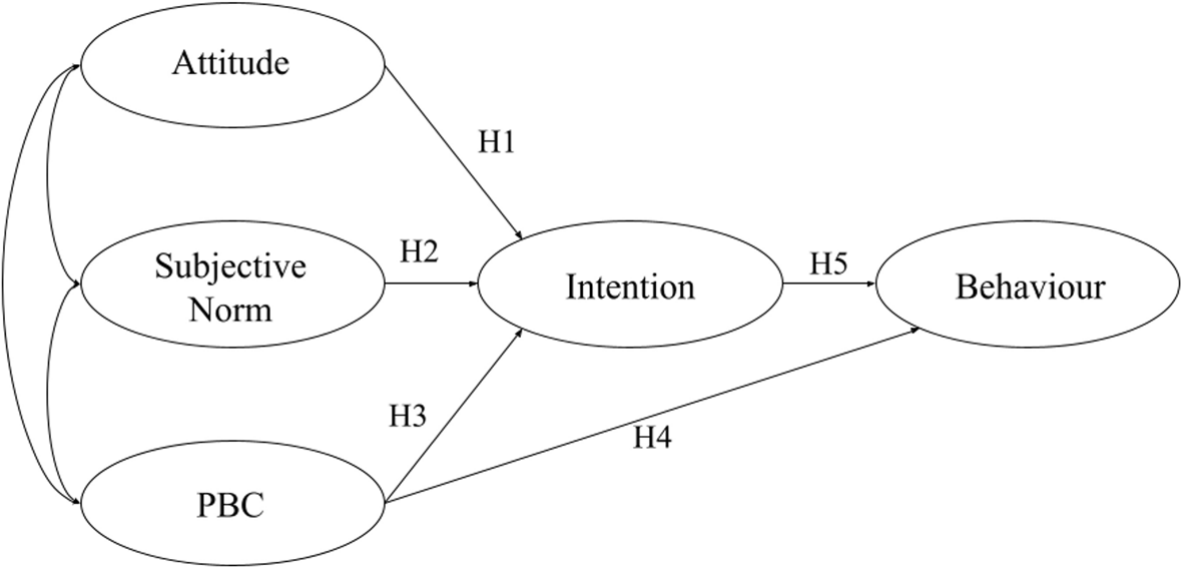
Modeling the latent variables of the TPB whose hypotheses are tested using SEM

The TPB has been widely used, and the results show that attitudes, SN, and PBC account for a large portion of the variation in a variety of behaviors [38]. According to the study conducted on native American youth, SN and PBC predict 30% of the variance in intention to adopt healthier eating behaviors [39]. When TPB was applied to genetically modified food consumption, the explanatory power of the model was 44.4%, with attitude being the strongest predictor [40], whereas in organic food consumption studies, SN and attitude were the most important predictor of intention [41]. In this study, we aim to test the following hypotheses based on the TPB model as outlined in Fig. 1:

- H1: A favorable attitude predicts the intention to consume fast food.
- H2: SN predicts the intention to consume fast food.
- H3: PBC predicts the intention to consume fast food.
- H4: PBC predicts FFC.
- H5: Behavioral intentions predict FFC.

## Materials and methods

### Population, setting and study design

This cross-sectional study was conducted on college students in the Punjab region of Pakistan in 2020. Purposive sampling was utilized in this research. The objective of this study was to examine the FFC behaviors in college students based on TPB. The inclusion criteria of the study were students above the age of 16 years old, which is the average age for college students in Pakistan. Any college students who were on a special diet such as a nutrition intervention diet and those consuming medications for gastrointestinal diseases were excluded from the research study. A total number of 220 college students were recruited for this study.

### Questionnaire development

This study used Mirkarimi and colleagues’ questionnaire to investigate FFC behavior among college students in Pakistan using TPB [42]. The questionnaire was divided into six categories (demographics, FFC pattern, intention for consuming fast food, attitudes towards FFC, SN, and PBC), included 52 questions, and a 4-point Likert scale answers options (1: not at all, 2: a little, 3: somewhat, 4: very much) [42]. This questionnaire was originally tested and subsequently applied in Iran. Face validity was examined by three specialists in health education and two specialists in nutrition, and its content validity was examined by seven specialists in health education and two specialists in nutrition [42]. Pakistan has similar teaching and learning cultures, primary, secondary, and further education, as well as health care settings to those in Iran [43, 44]. Therefore, the use of this questionnaire is considered appropriate for this research in Pakistan.

### General characteristics

The first section of the questionnaire includes questions about height, weight, BMI, mother’s education, father’s education, interest in health, interest in weight control, and with whom they usually eat. The second section was about patterns of FFC. It contains questions about the type of fast food consumed the most (hamburger, French fries, pizza, doughnuts, and fried chicken), reasons for consuming fast food, with whom they enjoy eating fast food, and places where they eat fast food.

### Constructs of the TPB

This part contained 39 questions regarding the four constructs of the TPB (BI, attitudes, SN, and PBC). These questions were assessed by using a 4-point Likert scale (1: not at all, 2: a little, 3: somewhat, 4: very much).

The first section explores the BI to consume fast food by asking five questions about different fast-food items. Questions are asked about the inclination/intention of consuming hamburgers, French fries, pizza, fried chicken, and donuts in a month such as ‘I intend to eat hamburgers in a month’. The scores for measuring BI ranged from 5 to 20.

The second section studies attitudes towards FFC by asking 12 questions about familiarity, health status, vital nutrients, taste, the attractiveness of the environment, fast food portion size, energy and salt content of the food, fat content within the food, beliefs about becoming overweight after eating fast food, and the relationship between fast food and body weight. The scores for measuring attitudes ranged from 12 to 48.

The third section investigates SN by determining normative beliefs and motivation to comply. It asks nine questions about family, teachers, and friends' impact on FFC beliefs such as “I think that my friends would like my fast-food consumption” or “I follow my family’s beliefs”. The scores for measuring SN ranged from 9 to 27.

The fourth section explores PBC towards FFC by asking 13 questions about fast food use. These questions are based on the perception of how difficult it is to perform a behavior. This section includes questions about their thinking about eating fast food even if few outlets are near, considering it difficult to meet at different places other than fast food stores, eating fast food even if on diet or having to wait for long, eating fast food even if few advertisements are on TV or if fast food stores offer few sale promotions, and about considering a change in FFC behavior to improve health. The scores for measuring PBC ranged from 13 to 52.

### Data collection

Detailed information about the purpose of the research project, the questionnaire, and the use of data were explained in person by the researcher to the students in the classroom and by phone to parents of students under the age of 18 years old. Informed consent was obtained in person from all participants who were interested in participating and met the inclusion criteria. Parents of the 42 students who were under the age of 18 years old provided a verbal consent by phone and sent the signed written consent to the school with their children. Questionnaires were then provided, and explanations were offered if the participants required more information.

Physical copies of the questionnaire were distributed to the participants who completed the informed consent, and a brief explanation of the task was given by the researcher. Upon completion of the questionnaire, participants were asked to put it in an envelope and drop it in the provided data collection box. Following collection, all data were anonymized and the participants were allowed to withdraw from the study at any point in time. Anthropometric measurements were offered to participants who did not know their exact height and weight.

### Structural equation modeling analysis

Structural equation modeling (SEM) is used to evaluate multivariate causal relationships, as well as direct and indirect effects between variables for an a priori latent variable model of interest [45]. It is a combination of confirmatory factor analysis and path analysis. Path analysis finds causal relationships among variables in a defined path, and confirmatory factor analysis estimates abstract traits by testing theoretical assumptions against collected data [45, 46]. SEM assumes linearity and unidimensionality among its variables, differentiating it from regression modeling. The causal diagrams produced by SEM also give rise to nonparametric structural equations being causally interpreted [46]. SEM measures the influence of multiple variables on one another simultaneously with the relationships defined by a specified model [45]. In this study, SEM was applied to test the hypothesized TPB model outlined previously in Fig. 1.

Goodness-of-fit indices are used to test how well-observed data fit the theory-based model [47]. Indices used to assess model fit were chi-square (χ2), Tucker-Lewis index (TLI), Comparative fit index (CFI), and the root mean square error of approximation (RMSEA) as recommended by Garver and Mentzer [48]. TLI compares a proposed model’s fit to a null model and measures parsimony by comparing the degrees of freedom of the proposed model to the null model. CFI is a noncentrality index value that accounts for sample size effects. RMSEA measures the difference between observed and estimated covariance matrix values per degree of freedom and is measured in terms of the population rather than the sample [45]. A low chi-square value demonstrates no significance in the difference between the specified hypothesized model and the data, thus demonstrating a good fit. However, since the chi-square test is sensitive to sample size, especially when the sample is larger than 200, a ratio of the chi-square value to degrees of freedom is used to evaluate goodness-of-fit [45]. A good model fit is obtained when TLI > 0.90, CFI > 0.90, RMSEA < 0.05 [45], and when the ratio of the chi-square value to degrees of freedom is less than 3 [49].

### Statistical analyses

SEM analysis was performed using SPSS 29.0.0.0 and SPSS AMOS 26. Patterns of FFC, intention for consuming fast food, attitude toward FFC, the SN for FFC, and PBC toward FFC were identified as the latent variables of the TPB model, with each questionnaire item per latent category being the observed variables through which latent variables become measurable.

Descriptive statistics such as mean, standard deviation, frequency, and percentages were applied along with an independent t-test and chi-square. All statistical analysis was performed by using SPSS 17.0 software. T-test was applied for the assessment of attitude, SN for FFC, and PBC towards FFC. Pearson Correlation coefficient and multiple linear regression were used to investigate the association between all the constructs of the TPB and FFC. FFC was the dependent variable in this study while the general characteristics, constructs of the TPB intention, SN, behavior, and attitudes related to FFC were identified as the independent variables.

## Results

### Descriptive characteristics

A total of 250 questionnaires were distributed to the research participants, of which 30 surveys were incomplete and removed from the data analysis, leaving 220 college students (97 male and 123 female) included. Measurements of height, weight, and BMI were taken for all participants. According to the BMI classifications, 129 students (68 male, 61 female) had normal BMI, 62 students (17 male, 45 female) were underweight, 20 students (8 males, 12 females) were overweight, and nine students (4 males and 5 females) were obese. The chi-square results showed a statistically significant association between gender and BMI categories (13.2 (*p* = 0.004). Ninety students had a high interest in health, while 68 students showed an average interest. Overall, most students were interested in controlling and maintaining their weight to a healthy BMI. The association between gender and the question “who do you usually eat with” was also statistically significant with a *p*-value of 0.015. From the total sample, 137 students (56 males and 81 females) reported eating with their whole family. Details of the students’ characteristics and chi-square results are presented in Table 1.

**Table 1:** Participants’ characteristics and chi-square results

**Variables**	**Variance levels**	**Gender**			chi-square test (*p*-value)
		Total (%)	Male n (%)	Female n (%)	
**Body Mass Index (BMI)**	Underweight (less than 18.5)	62 (28.2)	17 (27.42)	45 (72.58)	13.2 (*p* = .004)
	Normal (between 18.5 and 24.9)	129 (58.2)	68 (52.71)	61 (47.29)	
	Overweight (between 25 and 29.9)	20 (9.1)	8 (40.00)	12 (60.00)	
	Obese (more than 29.9)	9 (4.1)	4 (44.44)	5 (55.56)	
**Mother Education**	Primary School	30(13.6)	18(18.6)	12(9.8)	8.35 (*p* = .080)
	Secondary School	41(18.6)	17(17.5)	24(19.5)	
	High School	73(33.2)	24(24.7)	49(39.8)	
	Bachelors or above	62(28.2)	30(30.9)	32(26.0)	
	None	14(6.4)	8(8.2)	6(4.9)	
	Total	220(100)	97(100)	123(100)	
**Father Education**	Primary School	20(9.1)	11(9)	9(7.3)	1.347 (*p* = .853)
	Secondary School	40(18.2)	18(18.6)	22(17.9)	
	High School	72(32.7)	32(33)	40(32.5)	
	Bachelors or above	76(34.5)	31(32)	45(36.6)	
	None	12(5.5)	5(5.2)	7(5.7)	
	Total	220(100)	97(100)	123(100)	
**Interest in health**	very little	10(4.5)	4(4.1)	6(4.9)	3.94(*p* = .415)
	little	10(4.5)	5(5.2)	5(4.1)	
	average	68(30.9)	35(36.1)	33(26.8)	
	much	42(19.1)	20(20.6)	22(17.9)	
	very much	90(40.9)	33(34.0)	57(46.3)	
	Total	220(100)	97(100)	123(100)	
**Interest in weight control**	Very little	31(14.1)	13(13.4)	18(14.6)	5.622 (*p* = .229)
	Little	15(6.8)	7(7.2)	8(6.5)	
	Average	37(16.8)	11 (22.7)	26 (12.20)	
	Much	38(17.3)	18(18.6)	20(16.3)	
	very much	99(45)	37(38.1)	62(50.4)	
	Total	220(100)	97(100)	123(100)	
**Who do you usually eat with**	Whole family	137(62.3)	56(57.7)	81(65.9)	10.45 (*p* = .015)
	Some members of a family	43(19.5)	18(18.6)	25(20.3)	
	Alone	29(13.2)	13(13.4)	16(13)	
	friends	11(5.0)	10(10.3)	1(0.8)	
	Total	220(100)	97(100)	123(100)	

### Patterns of FFC

The pattern of FFC among college students is presented in Table 2. The association between gender and type of fast food consumed was statistically significant (*p* = 0.008) with a chi-square of 13.92; females tend to eat more French fries and pizza whereas fried chicken consumption was more by males. Furthermore, there was a statistically significant association between gender and the reasons for FFC with a p-value of 0.005 and a chi-square value of 15.040. For example, 73 female students reported that they eat fast food on special occasions, whereas more male students eat fast food when they are with friends. Most students consume fast food with their friends (*n* = 102) and the association between this category and gender was statistically significant with a p-value of 0.007 and a chi-square value of 13.93. More students overall, especially females, considered eating fast food as a snack rather than viewing it as a proper meal.

**Table 2:** Patterns of fast-food consumption among college students

**Variables**	**Variance levels**		**Gender**		chi-square test (*p*-value)
		Total (%)	Male N (%)	Female N (%)	
Which of the following food do you consume the most	Hamburger	26(11.8)	13(13.4)	13(10.6)	13.92 (*p* = .008)
	French fries	66(30.0)	31(32)	35(28.5)	
	Pizza	77(35.0)	22(22.7)	55(44.7)	
	Fried chicken	48(21.8)	29(29.9)	19(15.4)	
	Doughnuts	3(1.4)	2(2.1)	1(0.8)	
Reasons for consuming fast food	Alone at home	20(9.1)	12(12.4)	8(6.5)	15.040 (*p* = .005)
	special days	106(48.2)	33(34)	73(59.3)	
	meeting friends	41(18.6)	25(25.8)	16(13)	
	when hungry	31(14.1)	16(16.5)	15(12.2)	
	out of habit	22(10.0)	11(11.3)	11(5.9)	
	Total	220(100)	97(100)	123(100)	
With whom do you enjoy eating fast food?	Parents	42(19.1)	13(13.4)	13(10.6)	13.93 (*p* = .007)
	Siblings	49(22.3)	31(32)	35(28.5)	
	Friends	102(46.4)	22(22.7)	55(44.7)	
	Alone	22(10)	29(29.9)	19(15.4)	
	Relatives	5(2.3)	2(2.1)	1(0.8)	
	Total	220(100)	97(100)	123(100)	
What do you consume fast food as?	As meal	107(48.6)	52(53.6)	55(44.7)	1.72 (*p* = .190)
	As snack	113(51.4)	45(46.4)	68(55.3)	
	Total	220(100)	97(100)	123(100)	
Place where you eat fast food	Near home	71(32.3)	30(30.9)	41(33.3)	2.833 (*p* = .418)
	Near school	23(10.5)	8(8.2)	15(12.2)	
	Downtown	32(14.5)	18(18.6)	14(11.4)	
	Others	94(42.7)	41(42.3)	53(43.1)	
	Total	220(100)	97(100)	123(100)	

### Mean scores of the constructs of the TPB among college students

The mean scores of the BI and attitudes were higher for female students, while SN and PBC mean scores were higher for males. However, these results were not statistically significant (*p* > 0.05). The only statistically significant association was between the mean of PBC and gender (*p* = 0.025). Details of the mean score and statistical testing of the constructs of the TBP are presented in Table 3.

**Table 3:** Mean Scores and statistical testing of the constructs of the theory of planned behavior

**Variables**	**Total** M ± SD	**Gender**		t-value	*p*-value
		Male M ± SD	Female M ± SD		
**Behavioral Intention**	10.95 ± 5.336	10.71 ± 5.06	11.09 ± 5.55	-.850	0.396
**Attitude**	35.77 ± 13.21	34.93 ± 11.75	35.5 ± 13.6	-.33	0.740
**Subjective Norms**	28.29 ± 11.5	29.54 ± 12.23	27.79 ± 10.78	-.483	0.630
**Perceived Behavioral Control**	31.73 ± 14.47	33.88 ± 13.8	30.06 ± 11.36	2.252	0.025

### Correlation among variables of the TPB and FFC

Table 4 presents the Pearson coefficient of correlation among FFC frequency, BI, attitude, SN, and PBC. This highlights the strength of the association between the variables. Some variables were significantly correlated with each other, particularly. Notably, FFC was highly correlated with BI (0.767), whereas the other constructs were moderately correlated with each other.

**Table 4:** Pearson coefficient of correlation among fast-food consumption frequency, intention, attitude, subjective norm, and perceived control

Variables	FFC frequency	Behavioral Intention	Attitude	Subjective Norm	Perceived control
FFC frequency	1				
Behavioral Intention	.767^**^	1			
Attitude	.442^**^	.304^**^	1		
Subjective Norm	.406^**^	.384^**^	.430^**^	1	
Perceived control	.489^**^	.337^**^	.496^**^	.450^**^	1

^**^ *p* < .01, Weak correlation = 0.1–0.29, Moderate = .30–0.49, Strong = .50–1.00

### Multiple regression on (BI)and FFC

Multiple regression analysis was conducted to investigate the relationship between different constructs of the TPB. This analysis shows there was no statistically significant effect of attitude on BI but there was a significant effect of SN (β = 0.262, *p* < 0.001) and PBC (β = 0.164, *p* < 0.001) on BI. Thus, SN and PBC are predictors of BI. Table 5 presents a summary of the Multiple Regression analysis demonstrating the effect of Attitude, SN, and PBC on BI. Moreover, the effect of BI and SN on FFC was calculated and the results were summarized in Table 6 which presents a summary of Multiple Regression: Effect of BI, SN on Frequency of FFC. R^2^ = 0.603 shows that 60% of the variation in FFC is attributable to BI and SN. There was a significant association between BI (β = 0.717, *p* < 0.001) and SN (β = 0.131, p.005), and FFC, which suggests BI and SN are strong predictors of FFC.

**Table 5:** Summary of Multiple Regression: Effect of Attitude, Subjective Norm, and Perceived Control on Behavioral intention

**Constructs**	**Unstandardized Coefficients**		**Standardized Coefficients**	t-value	*P*-value	R^2^
	B	Std. Error	Βeta			
Attitudes	.049	.033	.110	1.51	.133	.190
Subjective Norm	.152	.041	.262	3.68	< .001*	
Perceived control	.055	.025	.164	2.22	.028*	

^*^ *p* < .05, (BI)as a dependent variable

**Table 6:** Summary of Multiple Regression: Effect of Behavioral intention, Subjective Norm on Frequency of fast-food consumption

**Constructs**	**Unstandardized Coefficients**		**Standardized Coefficients**	t-value	*p*-value	R^2^
	B	Std. Error	Βeta			
Behavioral Intention	.401	.026	.717	15.47	< .001	.603
Subjective Norm	.043	.015	.131	2.838	.005*	

^*^ *P* < .05. FFC is a dependent variable

### SEM analysis and hypothesis testing

Cronbach’s alpha measures the internal consistency of a construct, with its values ranging from 0 to 1. Internal consistency assesses the degree to which all items in a construct measure the same concept. As the alpha value increases, the proportion of the test score attributable to error decreases [50]. The alpha value is sensitive to the correlation between items, dimensionality, and the length of the questionnaire. Greater correlation between items decreases the alpha value, alpha values are only applicable when test items are unidimensional, and the alpha value is directly proportional to questionnaire length per construct [50]. For constructs with less than ten items, a Cronbach’s alpha value greater than 0.5 demonstrates good reliability. For constructs with ten or more items, a Cronbach’s alpha value greater than 0.7 demonstrates good reliability [51, 52]. In Table 7, the constructs of attitude (0.585)*,* intention (0.445)*,* and behavior (0.022) have low Cronbach’s alpha values, indicating that the items used to measure these constructs have low reliability. The constructs SN (0.616) and PBC (0.783) have acceptable Cronbach’s alpha values given the number of items, demonstrating that the items used to measure the given constructs have good reliability.

**Table 7:** Descriptive statistics; Cronbach’s α, mean score, and standard deviation (SD)

Construct item	Mean (SD)
**Attitude (α = 0.585)**	
Fast food is familiar to me	3.174 (1.012)
I think that fast food is not good for health	3.356 (0.973)
I think that fast food can provide all vital nutrients for us	2.178 (0.934)
I think that fast food is delicious	3.584 (0.752)
I think that fast-food stores provide an attractive environment	2.890 (1.026)
I think that fast-food stores are clean	2.479 (0.945)
I think that fast food is clean and safe	2.315 (0.907)
I think that fast food portions are large enough to feel full	2.872 (0.987)
I think that fast food has a lot of salt	2.758 (0.972)
I think that fast food has a lot of fat	3.443 (0.857)
I think that consuming fast food will make me fat	3.425 (0.855)
I think that fast food has a lot of calories	3.393 (2.943)
**Subjective norm (α = 0.616)**	
Motivation to comply	1.995 (1.029)
Normative Belief	2.548 (0.841)
Motivation to comply × Normative belief	2.251 (0.998)
**Perceived behavioral control (α = 0.783)**	
I can eat fast food even if fewer fast-food stores are near	2.493 (0.955)
I think that meeting friends at places other than fast food stores would be difficult	2.616 (1.092)
I can eat fast food even while I am on diet	2.352 (1.066)
I can eat fast food even if I have to wait for a long time	2.475 (1.102)
I can eat fast food even if the fewer advertisement for fast food is on TV, the internet, etc	2.448 (1.054)
I can eat fast food even if they offer a few sale promotions	2.438 (1.022)
I think that changing my fast food consumption behaviors for health would be difficult	2.580 (1.056)
I think that using places other than fast food stores for special occasions such as birthdays would be difficult	2.479 (1.020)
I think that changing my fast food consumption behavior is difficult because I have eaten them from very young ages	2.758 (2.250)
I can eat fast food even if I get nutrition education using multimedia (e.g. website, video clip) rather than a basic lecture or brochure	2.306 (0.910)
I can eat fast food even if I get continued nutrition education	2.283 (0.944)
I can eat fast food even if I learn how to quickly prepare a simple meal	2.333 (1.019)
I can eat fast food even if I get nutrition education about the impact of fast food on health (e.g. calories, nutrient content)	2.223 (0.958)
**Intention (α = 0.445)**	
I intend to eat hamburgers in a month	1.799 (0.960)
I intend to eat French fries in a month	1.447 (0.894)
I intend to eat a pizza in a month	1.361 (0.600)
I intend to eat fried chicken in a month	1.379 (0.589)
I intend to eat doughnuts in a month	1.890 (0.661)
**Behavior (α = 0.022)**	
Which of the following food do you consume the most?	2.712 (0.983)
Reasons for consuming fast food	2.763 (1.244)
With whom do you enjoy eating fast food?	2.548 (0.982)
What do you consume fast food as?	1.530 (0.544)
Place where you eat fast food	2.676 (1.317)

Table 8 lists the goodness-of-fit indices for the data when originally fitted to the hypothesized model, and after modification to improve fit. The collected data do not demonstrate adequate fit with the TPB hypothesized model due to the values of TLI, CFI, and RMSEA not meeting the minimum requirements. The model was modified by removing measured variables with low factor loadings with values less than 0.5 [53]. A total of eight measured variables with low factor loadings were removed. The model was further modified using modification indices produced by SPSS AMOS to account for residual correlations [53]. A total of 34 residual correlations were added to the model. Despite the modification, the data do not meet all the requirements to demonstrate a good fit with the theoretical model. While RMSEA improves and meets the minimum requirement for the data to have a good fit with the modified hypothesized model, TLI, and CFI values are below 0.90. Therefore, the data do not demonstrate a good fit with the theoretical model. As a result, the unidimensionality of the variables cannot be assessed [45], as well as coefficients between latent variables to assess tenets of the hypothesized TPB model in Fig. 1.

**Table 8:** Fit indices of hypothesized and modified TPB models

Model	Degrees of freedom	Chi-squared	CMIN/DF	TLI	CFI	RMSEA
Hypothesized Model	657	1489.794	2.268	0.562	0.590	0.076
Modified Model	363	557.488	1.536	0.863	0.885	0.050

## Discussion

This study examined the state of FFC among male and female college students in Pakistan. Results of the SEM analysis demonstrate incompatibility between the data collected and the theoretical TPB model it was fitted with. Therefore, the five hypotheses of the TPB model outlined in Fig. 1 cannot be tested, and the results of the SEM analysis cannot be interpreted due to the poor fit of the model with the data. Nevertheless, descriptive statistics and regression analyses allow for observations of relationships between the TPB constructs of interest.

The findings of our multiple regression analysis indicate that TPB constructs (SN and BI) positively manifested the behavior of FFC. Most participants (58.2%) had normal BMI, while 9.1% and 4.1% were overweight and obese, respectively. The percentage of overweight students is higher than the reported percentage in similar studies. For example, results from a study conducted on middle (junior/primary) school students in Seoul showed 1.4% of participants were in the overweight category [1]. Our study also construed female students (46.3%) as being more concerned about their weight compared to male students (34%). This was an interesting finding as a similar study conducted in the USA by Hyun-sun Seo noted that male students were more concerned about their weight and health in the American college culture than female students [54].

In this study, most female students considered consuming fast food as snacks while the male students considered it as meals, which is in agreement with several studies [54, 55]. This may have some intercorrelation with the idea that female college students reflect on their daily calorie intake and tend to control their weight. Several studies suggest that nutritional education programs on energy content in fast food can help prevent the overuse of fast food such as snacks. This study also showed that students mostly consumed fast food near their homes (32.3%) and when out with friends (42.7%). Some students also reported consuming fast food when shopping or socializing in urbanized areas of the town and city center. Hyun-sun Seo's study also suggested that most college students choose to eat and socialize using fast-food restaurants in the city rather than playing at home or school [54].

Our study presented the mean score of BI being 10.95 ± 5.336 for FFC. The FFC was highly correlated with BI based on the analysis of the Pearson correlation of coefficient. These findings indicated that most students understood fast food was not good for their health and appreciate the risks associated with a fast-food diet. However, some studies suggested that nutritional knowledge related to the harmful effects of FFC is not sufficient and this could affect the participant’s attitudes and behaviors toward their food choices [56, 57]. Considering the data, nutrition intervention programs could provide information to the students about the harmful effects of fast food and may have some impact on their FFC. However, this suggestion is yet to be investigated in future studies.

This study showed that friends have the most impact on the students' FFC in the category of SN. The total sum of the mean of friends was higher when compared with family and teachers. The result also suggested the education system should list friends as a target group that can affect the consumption of fast food. The same conclusion was interpreted by other studies with similar results [6, 54].

The PBC was moderate with a score of 31.73 out of 52. Male students reported a higher level of PBC (33.88) than female students (30.06). This implies that external factors do not affect the consumption of fast food in male college students as much as in female students. A similar study by Kamal Mirkarimi showed similar results of male college students having higher scores of PBC [55].

When the Pearson coefficient of correlation was applied among constructs of TBP and FFC, FFC was highly correlated with BI. Attitude, SN, and PBC were moderately correlated with FFC. However, we were unable to test the hypotheses of these TPB models due to the poor fit of the model with the data and the incompatibility between the data collected and the theoretical TPB model in the SEM analysis. In a similar study, the intention to consume fast food was observed as a strong predictor of the frequency of FFC [58]. Regression analysis showed that SN and PBC are the strongest predictors of BI. However, some studies have concluded different results such as Yarmohammai and colleagues and Dunn and colleagues who reported attitude was the most significant predictor of BI [27, 59]. Concerning FFC, SN, and BI were observed as the strongest predictors of FFC with an R^2^ of 0.603. This result is consistent with a study by Ebadi and colleagues, which suggests BI is a strong predictor of FFC [60]. From these findings, this study can suggest that strong predictors should be targeted during intervention planning for changing the dietary habits of the students.

A study conducted in a high school in Iran showed that TPB explained the variance with a rate of 25.7% of intentions with attitude as the strongest predictor (B = 0.31, *P* < 0.001) and SN as the weakest factor (B = 0.29, *P* < 0.001) [61]. Another study conducted in Seoul used TPB to assess the factors that influence FFC [1]. It concluded that TPB demonstrated FFC behaviors with a relatively high R^2^ around 0.6. Using multiple regressions, it has been observed that FFC was meaningfully related to BI (b = 0.61, *P* < 0.001) and PBC (b = 0.19, *P* < 0.001). Furthermore, BI was expressively related to SN (b = 0.15, *P* < 0.01) and PBC (b = 0.56, *P* < 0.001) [1]. When gender differences were investigated using TPB, it was acknowledged that boys’ eating behavior was predicted by SN and PBC whereas the eating behavior of girls was predicted by attitudes, SN, and self-efficacy [62]. Another study stated that the reasons given by students for eating fast food were because it was inexpensive, as well as the enjoyment of its taste, and how the participants enjoyed eating fast food with their friends [54]. A greater number of male students stated they participated in eating at fast food restaurants compared to female students [54].

The data suggest that such social behaviors and consumption of westernized fast food impact traditional culture whereby meals would always be consumed by a family at home. Furthermore, changing tastes toward western fast-food options are increasing due to the cost-effectiveness and ability to consume food quickly.

### Strengths and limitations

To the best of our knowledge, this is the first study to investigate the behavioral factors that impact college students' FFC in Pakistan by applying TPB. It uses a comprehensive questionnaire and collects a significant amount of data related to the association between TBP constructs and FFC. To analyze the data this study uses comprehensive statistical tests that include descriptive statistics, chi-square, t-test, Pearson Correlation coefficient, multiple linear regression, and SEM. Nevertheless, this study has some limitations. It included only a small sample of college students and did not include some factors such as the amount of energy intake, and macro and micronutrients that may have a direct impact on increasing the risk of developing FFC-related diseases. Therefore, it is recommended to repeat this study with a larger sample that includes these factors.

Limitations that arise from the SEM analysis are evident in the low internal consistency observed for each construct item. If observed variables associated with a given latent variable are unable to provide the latent variable with a measurable dimension, it lowers Cronbach’s alpha value, thus demonstrating that the linear relationship from the observed variable to the latent is not applicable. Moreover, the study is affected by the common method bias [63] due to adapting Mirkarimi and colleagues’ questionnaire without modification; both positively and negatively connotated questions use the same scales, therefore limiting the ability to have a uniform collection of data and introducing common variation. This is evident in items such as “I think fast food is not good for health” and “I think fast food can provide all vital nutrients for us” used to measure the latent variable of Attitude. Both items use the 4-point Likert scale. By using items with opposing connotations on the same scale, the questionnaire gives rise to common variations among the measured variables for a latent variable [63].

Moreover, the SEM analysis is limited by the model size effect caused by having too many observed variables thus having a large number of degrees of freedom. This results in a poor approximation of the chi-squared distribution, preventing the chi-square value from demonstrating the goodness of fit due to the chi-square test rejecting the most correct models that fit the data, thus giving a high chi-square value and low p-value (< 0.001) [64]. In SEM analysis, increasing the number of indicators leads to a poor fit of the data with the specified model. The questionnaire employed in this study has a total of 38 items corresponding to the constructs of TPB; it is recommended that for samples where N is approximately 200, 30 or fewer items should be used, otherwise N ≥ 500 is recommended for larger models with more measured items [65].

### Implications for future practice and research

The results of this study can add to the body of knowledge on nutrition in college students and support program planners who want to improve the health and wellness of their students. This could encourage young people to understand the harmful effects of excessive FFC on health and well-being. Based on the findings of this research, we support the suggestion that nutrition education programs targeting family and friends of college students would help minimize the consumption of fast food [32]. Furthermore, this research has become confined through its cross-sectional nature. Participants consume fast food whilst being college students and it appears that this is consistent and incongruent with research discussed in other countries. Other research studies measuring the influence of acceptance and rejection of FFC would be useful in furthering the knowledge in this field. Additionally, understanding why college students partake in FFC while knowing the risks would be a useful study, potentially examining the lived experience of these students using a phenomenological methodology. Colleges working with healthcare and governmental institutions planning a nutrition education program should consider social sciences and social norms when planning any behavioral changes, as SN and PBC seem to be suggested in the study.

Taking into consideration the limitations of this study, future research could aim to test hypotheses based on the outcome of this research, such as investigating whether external factors affect the FFC more in females than males. However, we recommend that SEM analysis to test the hypothesis related to the constructs of TPB must be considered at the design stage of the data collection by limiting the number of indicators to facilitate a good fit of the data with the specified model. Furthermore, future research should include several age groups and assess other factors related to the nature of fast food that may have a direct impact on health. Future research should associate eating habits and FFC with other types of objective and measurable clinical data, allowing researchers to prove not only the existence of a link between these factors and a variety of non-communicable diseases but also to quantify the magnitude of the link.

## Conclusions

The use of SEM analysis in testing the hypothesis related to the constructs of TPB requires limiting the number of indicators (≤ 30) or a greater sample size (N ≥ 500) to facilitate a good fit of the data with the specified model. The study findings suggest that Pakistani college students' decision to consume fast food is mainly influenced by their friends and the increased popularity of fast food in Pakistan, despite their knowledge of its unhealthy nature. Therefore, educational programs aimed at reducing FFC should focus on specific harmful effects of fast food, rather than just general knowledge of its negative impacts. The study findings also indicate that among the constructs of TPB, SN, and BI are the strongest predictors of FFC. As such, health organizations and higher education institutions can use our data to develop targeted interventional health strategies. This study also offers data that could be useful in future sociological, epidemiological, psychological, and nutritional research.

## Supplementary Information

**Additional file 1.**

## Data Availability

The datasets used and/or analyzed during the current study are available from the corresponding author upon reasonable request.

## Abbreviations

BMI: Body Mass Index
BI: Behavioral Intention
χ2: Chi-square
CFI: Comparative fit index
FFC: Fast Food Consumption
RMSEA: Root mean square error of approximation
SEM: Structural Equation Modeling
SN: Subjective Norm
PBC: Perceived Behavioral Control
TPB: Theory of Planned Behavior
TLI: Tucker-Lewis index

## References

[1] Seo HS, Lee SK, Nam S (2011). Factors influencing fast food consumption behaviors of middle-school students in Seoul: an application of theory of planned behaviors. Nutr Res Pract.

[2] Nyachuba DG (2010). Foodborne illness: is it on the rise?. Nutr Rev.

[3] WHO. Overweight and Obesity. 2021. https://www.who.int/news-room/fact-sheets/detail/obesity-and-overweight. Accessed 20 Dec 2022.

[4] Manuel S-S, Luis G-M (2021). Nutrition, obesity and asthma inception in children The role of lung function. Nutrients.

[5] Lim HJ, Xue H, Wang Y. Global trends in obesity. In: Meiselman HL, editor. Handbook of Eating and Drinking: Interdisciplinary Perspectives. Springer International Publishing; 2020:1217–1235. 10.1007/978-3-030-14504-0_157.

[6] Didarloo A, Khalili S, Aghapour AA, Moghaddam-Tabrizi F, Mousavi SM (2022). Determining intention, fast food consumption and their related factors among university students by using a behavior change theory. BMC Public Health.

[7] Pase MP, Himali JJ, Beiser AS, Aparicio HJ, Satizabal CL, Vasan RS (2017). Sugar- and Artificially Sweetened Beverages and the Risks of Incident Stroke and Dementia: A Prospective Cohort Study. Stroke.

[8] Pereira MA, Kartashov AI, Ebbeling CB, Van Horn L, Slattery ML, Jacobs DR (2005). Fast-food habits, weight gain, and insulin resistance (the CARDIA study): 15-year prospective analysis. Lancet.

[9] Popkin BM, Adair LS, Ng SW (2012). Global nutrition transition and the pandemic of obesity in developing countries. Nutr Rev.

[10] Janssen HG, Davies IG, Richardson LD, Stevenson L (2018). Determinants of takeaway and fast food consumption: a narrative review. Nutr Res Rev.

[11] Memon NA (2016). Fast food: 2nd largest industry in Pakistan. Pakistan Food J.

[12] Baig AK, Saeed M (2012). Review of trends in fast food consumption. Eur J Econ Finance Adm Sci.

[13] Greenfield M (2018). India: How often do you eat fast food (any quick service restaurant) in any given week (on average)?. Hearst Magazine.

[14] Qasmi S, Akhtar U, Akram U, Raza H, Ali A, Rana T (2014). Fast food consumption Drift in Pakistani population. J Food Nutr Sci.

[15] Asif M, Aslam M, Altaf S, Atif S, Majid A (2020). Prevalence and Sociodemographic Factors of Overweight and Obesity among Pakistani Adults. J Obes Metab Syndr.

[16] Siddiqui M, Hameed R, Nadeem M, Mohammad T, Simbak N, Latif A (2018). Obesity in Pakistan; current and future perceptions. J Curr Trends Biomed Eng Biosci.

[17] Boek S, Bianco-Simeral S, Chan K, Goto K (2012). Gender and race are significant determinants of students' food choices on a college campus. J Nutr Educ Behav.

[18] Younas B, Khalid WA, Hassan MU (2021). Fast Food Consumption and Increased Caloric Intake Leading to Obesity a Survey among Pakistani Teenagers. Syst Rev Pharm.

[19] Gordon-Larsen P, Adair LS, Nelson MC, Popkin BM (2004). Five-year obesity incidence in the transition period between adolescence and adulthood: the National Longitudinal Study of Adolescent Health. Am J Clin Nutr.

[20] Woodward DR, Boon JA, Cumming FJ, Ball PJ, Williams HM, Hornsby H (1996). Adolescents' reported usage of selected foods in relation to their perceptions and social norms for those foods. Appetite.

[21] Dunn KI, Mohr PB, Wilson CJ, Wittert GA (2008). Beliefs about fast food in Australia: a qualitative analysis. Appetite.

[22] Branscum P, Sharma M (2012). Using the theory of planned behavior to predict two types of snack food consumption among midwestern upper elementary children: implications for practice. Int Q Community Health Educ.

[23] Ajzen I (1991). The theory of planned behavior. Organ Behav Hum Decis Process.

[24] Godin G, Kok G (1996). The theory of planned behavior: a review of its applications to health-related behaviors. Am J Health Promot.

[25] Ajzen I, Fishbein M. Understanding Attitudes and Predicting Social Behaviour. New Jersey: Prentice-Hall, Englewood Cliffs; 1980.

[26] Voss KE, Spangenberg ER, Grohmann B (2003). Measuring the hedonic and utilitarian dimensions of consumer attitude. J Mark Res.

[27] Dunn KI, Mohr P, Wilson CJ, Wittert GA (2011). Determinants of fast-food consumption. An application of the theory of planned behaviour. Appetite.

[28] Ajzen I (2020). The theory of planned behavior: Frequently asked questions. Hum Behav Emerg Technol.

[29] Nystrand BT, Olsen SO (2020). Consumers’ attitudes and intentions toward consuming functional foods in Norway. Food Qual Prefer.

[30] Montano DE, Kasprzyk D (2015). Theory of reasoned action, theory of planned behavior, and the integrated behavioral model. Health Behav Theory Res Pract.

[31] LaMorte WW (2019). Behavioral change models: The theory of planned behavior. Retrieved December.

[32] Ajzen I (2015). Consumer attitudes and behavior: the theory of planned behavior applied to food consumption decisions. Italian Rev Agric Econ.

[33] McDermott MS, Oliver M, Svenson A, Simnadis T, Beck EJ, Coltman T (2015). The theory of planned behaviour and discrete food choices: a systematic review and meta-analysis. Int J Behav Nutr Phys Act.

[34] Poirier MJ, Grépin KA, Grignon M (2020). Approaches and alternatives to the wealth index to measure socioeconomic status using survey data: a critical interpretive synthesis. Soc Indic Res.

[35] Rutstein SO, Staveteig S. Making the demographic and health surveys wealth index comparable. Rockville: ICF International; 2014.

[36] Brooks-Gunn J, Klebanov P, Liaw F-r, Duncan G. Toward an understanding of the effects of poverty upon children. In: Zuckerman BS, Fitzgerald HE, Lester BM. Children of poverty. New York: Routledge; 2021. p. 3–41.

[37] Malik SM, Bhutta ZA (2018). Reform of primary health care in Pakistan. Lancet.

[38] Armitage CJ, Conner M (2001). Efficacy of the theory of planned behaviour: A meta-analytic review. Br J Soc Psychol.

[39] Fila SA, Smith C (2006). Applying the Theory of Planned Behavior to healthy eating behaviors in urban Native American youth. Int J Behav Nutr Phys Act.

[40] Kim YG (2014). Ecological concerns about Genetically Modified (GM) food consumption using the Theory of Planned Behavior (TPB). Procedia Soc Behav Sci.

[41] Al-Swidi A, Mohammed Rafiul Huque S, Haroon Hafeez M, Noor Mohd Shariff  M (2014). The role of subjective norms in theory of planned behavior in the context of organic food consumption. British Food J.

[42] Mirkarimi K, Mansourian M, Kabir MJ, Ozouni- Davaji RB, Eri M, Hosseini SG (2016). Fast Food Consumption Behaviors in High-School Students based on the Theory of Planned Behavior (TPB). Int J Pediatr.

[43] Wang C, Tee M, Roy AE, Fardin MA, Srichokchatchawan W, Habib HA (2021). The impact of COVID-19 pandemic on physical and mental health of Asians: A study of seven middle-income countries in Asia. PLoS ONE.

[44] Esteghamati A, Khalilzadeh O, Anvari M, Meysamie A, Abbasi M, Forouzanfar M (2009). The economic costs of diabetes: a population-based study in Tehran. Iran Diabetologia.

[45] Hoe SL (2008). Issues and procedures in adopting structural equation modelling technique. J Quant Methods.

[46] Fan Y, Chen J, Shirkey G, John R, Wu SR, Park H (2016). Applications of structural equation modeling (SEM) in ecological studies: an updated review. Ecol Process.

[47] Taris T (2002). BM Byrne, Structural equation modeling with AMOS: Basic concepts, applications, and programming Mahwah NJ: Lawrence Erlbaum, 2001 0–8058-3322-6. Eur J Work Organ Psy.

[48] Garver MS, Mentzer JT (1999). Logistics research methods: employing structural equation modeling to test for construct validity. J Bus Logist.

[49] Kline RB (1998). Structural equation modeling.

[50] Tavakol M, Dennick R (2011). Making sense of Cronbach's alpha. Int J Med Educ.

[51] Pallant J. SPSS survival manual: A step by step guide to data analysis using IBM SPSS. 7th Ed. London: Routledge; 2020.

[52] Nunnally JC. Psychometric Theory. 2nd ed. New York: McGraw-Hill; 1978.

[53] Collier JE. Applied structural equation modeling using AMOS: Basic to advanced techniques. 1st Ed. New York: Routledge; 2020.

[54] Morse KL, Driskell JA. Observed sex differences in fast-food consumption and nutrition self-assessments and beliefs of college students. Nutrition research. 2009;29(3):173-9. 10.1016/j.nutres.2009.02.004. 10.1016/j.nutres.2009.02.004 19358931

[55] Shaban L, Alkazemi D. Trends in Fast-food Consumption among Kuwaiti Youth. Int J Prev Med. 2019;10:44. 10.4103/ijpvm.IJPVM_480_18. 10.4103/ijpvm.IJPVM_480_18 PMC6528418 31143418

[56] Baeck B, Lee Y (2006). Consumer's awareness and policies directions on food additives-focusing on consumer information. J Consum Stud.

[57] Aoki K, Shen J, Saijo T (2010). Consumer reaction to information on food additives: evidence from an eating experiment and a field survey. J Econ Behav Organ.

[58] Abraham S, Martinez M, Salas G, Smith J. College student’s perception of risk factors related to fast food consumption and their eating habits. J Nutr Hum Health. 2018;2(1). 10.35841/nutrition-human-health.2.1.18-21.

[59] Yarmohammadi P, Sharifirad GR, Azadbakht L, Morovati Sharifabad MA, Hassanzadeh A. Predictors of fast food consumption among high school students based on the theory of planned behavior. Health Sys Res. 2011;7(4).

[60] Ebadi L, Rakhshanderou S, Ghaffari M (2018). Determinants of fast food consumption among students of Tehran: Application of planned behavior theory. Int J Pediatr.

[61] Sharifirad G, Yarmohammadi P, Azadbakht L, Morowatisharifabad MA, Hassanzadeh A (2013). Determinants of Fast Food Consumption among Iranian High School Students Based on Planned Behavior Theory. J Obes.

[62] Fila SA, Smith C (2006). Applying the theory of planned behavior to healthy eating behaviors in urban Native American youth. Int J Behav Nutr Phys Act.

[63] Kock N (2015). Common method bias in PLS-SEM: A full collinearity assessment approach. Int J e-Collaboration (ijec).

[64] Deng L, Yang M, Marcoulides KM (2018). Structural equation modeling with many variables: A systematic review of issues and developments. Front Psychol.

[65] Shi D, Lee T, Maydeu-Olivares A (2019). Understanding the model size effect on SEM fit indices. Educ Psychol Measur.

