# Supplementary material for: Using the theory of planned behavior to predict factors influencing fast-food consumption among college students
Source: BMC Public Health. 2023 May 27;23:987. doi: 10.1186/s12889-023-15923-1 (PMC10224588; doi:10.1186/s12889-023-15923-1)
Supplement: Supplementary file 1 — Additional file 1. [file 12889_2023_15923_MOESM1_ESM.pdf]

QUESTIONNAIRE

Name: ______________________ **Gender:**______________________

Age: __________________ **Weight:** __________________

Height: ________________ **BMI:** ________________

QUESTION NO 1

DEMOGRAPHIC CHARACTERISTICS OF COLLEGE STUDENTS

Q#1	Mothers education	1. Primary School<br>2. Secondary school<br>3. High School<br>4. Academic Degree<br>5. None
Q#2	Fathers education	1. Primary School<br>2. Secondary school<br>3. High School<br>4. Academic Degree<br>5. None
Q#3	Do you have an interest in health?	1. Very much<br>2. Much<br>3. Average<br>4. Little<br>5. Very Little
Q#4	Do you have an interest in weight control?	1. Very much<br>2. Much<br>3. Average<br>4. Little<br>5. Very Little
Q#5	Who do you usually eat with?	1. Whole family

		2. Some members of the family<br>3. Alone<br>4. Friends<br>5. Relatives

QUESTION NO 2

PATTERNS OF FAST FOOD CONSUMPTION

Q#6	Which of the following food do you consume the most in a week?	1. Hamburger<br>2. French Fries<br>3. Pizza<br>4. Fried Chicken<br>5. Doughnuts
Q#7	Reasons for consuming fast food	1. When parents are not at home<br>2. Special days<br>3. Meeting Friends<br>4. When Hungry<br>5. Out of Habit
Q#8	To whom do you enjoy eating fast food?	1. Parents<br>2. Brothers/Sisters<br>3. Friends<br>4. Alone<br>5. Relatives
Q#9	What do you consume fast food as?	1. A meal<br>2. Snack
Q#10	Place where you eat fast food	1. Near Home<br>2. Near School<br>3. Downtown

		4. Others

QUESTION NO 3

INTENTION FOR CONSUMING FAST FOOD

Q#11	I am inclined to use hamburgers in a month	1. Not at all<br>2. A little<br>3. Somewhat<br>4. Very Much
Q#12	I am inclined to use French fries for a month	1. Not at all<br>2. A little<br>3. Somewhat<br>4. Very Much
Q#13	I am inclined to use a pizza in a month	1. Not at all<br>2. A little<br>3. Somewhat<br>4. Very Much
Q#14	I am inclined to use fried chicken in a month	1. Not at all<br>2. A little<br>3. Somewhat<br>4. Very Much
Q#15	I am inclined to use doughnuts in a month	1. Not at all<br>2. A little<br>3. Somewhat<br>4. Very Much

QUESTION NO 4

ATTITUDE TOWARD FAST FOOD CONSUMPTION

Q#16	Fast food is familiar to me	1. Not at all<br>2. A little<br>3. Somewhat<br>4. Very Much
Q#17	I think that fast food is not good for the health	1. Not at all

		2. A little<br>3. Somewhat<br>4. Very Much
Q#18	I think that fast food can provide all vital nutrients for us	1. Not at all<br>2. A little<br>3. Somewhat<br>4. Very Much
Q#19	I think that fast food is delicious	1. Not at all<br>2. A little<br>3. Somewhat<br>4. Very Much
Q#20	I think that fast food stores provide an attractive environments	1. Not at all<br>2. A little<br>3. Somewhat<br>4. Very Much
Q#21	I think that fast food stores are clean	1. Not at all<br>2. A little<br>3. Somewhat<br>4. Very Much
Q#22	I think that fast food is clean and safe	1. Not at all<br>2. A little<br>3. Somewhat<br>4. Very Much
Q#23	I think that fast food portions are large enough to feel full	1. Not at all<br>2. A little<br>3. Somewhat<br>4. Very Much
Q#24	I think that fast food has a lot of salt	1. Not at all<br>2. A little<br>3. Somewhat<br>4. Very Much

Q#25	I think that fast food has a lot of fat	1. Not at all<br>2. A little<br>3. Somewhat<br>4. Very Much
Q#26	I think that consuming fast food will make me fat	1. Not at all<br>2. A little<br>3. Somewhat<br>4. Very Much
Q#27	I think that fast food has a lot of calories	1. Not at all<br>2. A little<br>3. Somewhat<br>4. Very Much

QUESTION NO 5

SUBJECTIVE NORM FOR FAST FOOD CONSUMPTION

Q#28	Motivation to comply	1. I follow my family's beliefs<br>2. I follow the beliefs of teachers<br>3. I follow the beliefs of my friend	1. Not at all<br>2. A little<br>3. Somewhat<br>4. Very Much
Q#29	Normative Belief	1. I think that my familywould like my fast food consumption<br>2. I think that my teachers would likemy fast food consumption<br>3. I think that my friends would like my fast-food consumption	1. Not at all<br>2. A little<br>3. Somewhat<br>4. Very Much

Q#30	Motivation to comply × Normative belief	1. Family<br>2. Teachers<br>3. Friends	1. Not at all<br>2. A little<br>3. Somewhat<br>4. Very Much

QUESTION NO 6

PERCEIVED BEHAVIORAL CONTROL TOWARD FAST FOOD CONSUMPTION

Q#31	I can eat fast food even if fewer fast-food stores are near	1. Not at all<br>2. A little<br>3. Somewhat<br>4. Very Much
Q#32	I think that meeting friends at places other than fast food stores would be difficult	1. Not at all<br>2. A little<br>3. Somewhat<br>4. Very Much
Q#33	I can eat fast food even while I am on diet.	1. Not at all<br>2. A little<br>3. Somewhat<br>4. Very Much
Q#34	I can eat fast food even if I have to wait for a long time	1. Not at all<br>2. A little<br>3. Somewhat<br>4. Very Much
Q#35	I can eat fast food even if the fewer advertisement for fast food is on TV, on the internet, etc.	1. Not at all<br>2. A little

		3. Somewhat<br>4. Very Much
Q#36	I can eat fast food even if they offer a few sale promotions	1. Not at all<br>2. A little<br>3. Somewhat<br>4. Very Much
Q#37	I think that changing my fast food consumption behaviors for health would be difficult	1. Not at all<br>2. A little<br>3. Somewhat<br>4. Very Much
Q#38	I think that using places other than fast food stores for special occasions such as birthdays would be difficult.	1. Not at all<br>2. A little<br>3. Somewhat<br>4. Very Much
Q#39	I think that changing my fast food consumption behavior is difficult because I have eaten them from a very young age	1. Not at all<br>2. A little<br>3. Somewhat<br>4. Very Much
Q#40	I can eat fast food even if I get nutrition education using multimedia (e.g website, video clip) rather than basic lecture or brochure	1. Not at all<br>2. A little<br>3. Somewhat<br>4. Very Much
Q#41	I can eat fast food even if I get continued nutrition education	1. Not at all<br>2. A little<br>3. Somewhat<br>4. Very Much
Q#42	I can eat fast food even if I learn how to quickly prepare a simple meal	1. Not at all<br>2. A little<br>3. Somewhat<br>4. Very Much

Q#43	I can eat fast food even if I get nutrition education about the impact of fast food on health (e.g. calories, nutrient content).	1. Not at all<br>2. A little<br>3. Somewhat<br>4. Very Much
